# Discharge Body Mass Index, Not Illness Chronicity, Predicts 6-Month Weight Outcome in Patients Hospitalized With Anorexia Nervosa

**DOI:** 10.3389/fpsyt.2021.641861

**Published:** 2021-02-25

**Authors:** Graham W. Redgrave, Colleen C. Schreyer, Janelle W. Coughlin, Laura K. Fischer, Allisyn Pletch, Angela S. Guarda

**Affiliations:** ^1^Department of Psychiatry and Behavioral Sciences, Johns Hopkins University School of Medicine, Baltimore, MD, United States; ^2^Children's National Medical Center, Clinical and Translational Science Institute, Washington, DC, United States

**Keywords:** inpatient, severe and enduring anorexia nervosa, treatment, outcomes, weight-restoration

## Abstract

Proposed treatments for severe and enduring anorexia nervosa (SE-AN) focus on quality of life, and psychological and social functioning. By de-emphasizing weight restoration as a priority, however, premature diagnosis of SE-AN may reduce potential for recovery. The present study assessed the effect of weight restoration, illness duration, and severity on treatment outcome 6 months after discharge from an intensive, meal-based behavioral treatment program. Participants included hospitalized adult women (*N* = 191) with AN or underweight other specified feeding and eating disorder (OSFED). Participants were characterized as short-term (ill <7 years; *n* = 74) or long-term ill (ill ≥ 7 years; *n* = 117). Compared with short-term ill, long-term ill patients were older, had lower lifetime body mass index (BMI), more prior admissions, and exhibited greater depression and neuroticism. Long-term vs. short-term ill patients gained weight at the same rate (~2 kg/wk) and were equally likely to be weight restored by discharge (>75% reached BMI ≥ 19 kg/m^2^ in both groups). At 6-month follow-up (*n* = 99), both groups had equivalent self-reported BMI, and depression, drive for thinness, body dissatisfaction, and bulimia scores. The only predictor of BMI ≥ 19 kg/m^2^ at follow-up was discharge BMI. The likelihood of a BMI ≥ 19 kg/m^2^ at follow-up was 5-fold higher for those with discharge BMI ≥ 19 kg/m^2^. Few studies of long-term ill inpatients with AN have examined the impact of full weight restoration on short-term outcomes. This study supports the therapeutically optimistic stance that, regardless of illness duration, hospitalized patients with AN benefit from gaining weight to a BMI ≥ 19 kg/m^2^.

## Introduction

Treatment of anorexia nervosa (AN) presents particular challenges to clinicians. Treatment is expensive, access is limited, and patient anxiety and ambivalence toward weight gain and behavior change can make treatment psychologically burdensome (1, 2). Furthermore, illness severity varies, from adolescents with recent onset AN, to adults disabled by the scar effect of many years of progressive functional impairment, physical morbidity, cognitive problems, and social isolation (3, 4). Protracted illness combined with multiple past treatment attempts and severe eating and weight control behaviors, can erode the hope of patients, family members, or clinicians, undermining future treatment expectations or recovery. Understanding factors that affect treatment outcome for the chronically ill patient with AN is thus of vital importance, especially in view of recent long-term follow-up studies that suggest recovery is possible even after decades of illness (5, 6).

Recovery from AN requires attainment of a healthy weight, and significant reduction or elimination of eating disordered behaviors and cognitions (7, 8). Attainment of a healthy weight is thus necessary if not sufficient for full recovery from AN, and low BMI at discharge from intensive treatment is the strongest known predictor of relapse and readmission for adults with AN (9–11). Even in outpatients, an analysis of five randomized controlled treatment trials for eating disorders, found weight restoration to a BMI > 19 kg/m^2^ the most efficient predictor of recovery at 1-year, for both adolescent and adult patients (12). Attainment of BMI ≥ 19 kg/m^2^ has been proposed as a threshold for full recovery from AN (7).

Several other factors across studies have been associated with outcome in AN, including illness duration, depressive and eating disorder psychopathology, motivation for treatment, interpersonal functioning, and early weight gain and behavior change in treatment (13). However, the findings with respect to these factors tend to be more mixed. In terms of illness duration, for example, some studies have found an association with outcome (14, 15), while others find no association of illness duration with outcome (16–18).

Illness duration has a central role in the construct of “severe and enduring” AN (SE-AN). Definitions of SE-AN vary, however most include measures of both illness duration and severity, as well as participation in past evidence-based treatments (4, 19–21). An early randomized controlled trial categorized patients with at least 7 years of illness as SE-AN (22), though a recent systematic review captures the lack of diagnostic precision and the breadth of terms applied to describe this group of patients (23). What level of chronicity equates to “severe and enduring” is unclear, and as few as 3 or as many as 10 years of illness have been used (3, 20, 24).

How to best measure illness severity in SE-AN is also unclear. Factors often considered include clinical characteristics, such as age, admission BMI, lifetime nadir BMI, number of hospitalizations, behaviors (e.g., purging), measures of eating disorder psychopathology, quality of life, or social and occupational functioning (13, 18). However, the one study that attempted empirical modeling of the SE-AN construct using a variety of clinical characteristics, including duration of illness and number of previous hospitalizations, found that these were not empirically useful in grouping patients (25).

The present descriptive study seeks to compare short- and long-term ill patients with AN on markers of severity and clinical course. We examined the effects of illness duration, illness severity, and weight restoration to a BMI ≥ 19 kg/m^2^, on the short-term (6-month) weight outcome of women with AN hospitalized in an integrated eating disorders inpatient-partial hospital behavioral program. Our primary hypothesis was that attainment of a BMI in the normal range at discharge would predict weight outcome at 6-month follow-up. In addition, we hypothesized that markers of severity, including illness duration, number of hospitalizations, and depressive and eating disorder psychopathology would predict weight outcome at 6-month follow-up.

## Methods

### Study Population

The study was approved by the Institutional Review Board of the Johns Hopkins University School of Medicine. All consecutive female first admissions to the Johns Hopkins Eating Disorders Inpatient-Partial Hospitalization Program between February 2003 and March 2015 with either AN or underweight other specified feeding and eating disorder (atypical AN with admission BMI <19; abbreviated OSFED) were invited to participate in a longitudinal study of treatment outcomes (*N* = 303). Participants (*N* = 191) provided verbal consent and completed a battery of self-report questionnaires at admission and 6 months after discharge. Institutional Review Board approval was also obtained for a chart review and abstraction of limited de-identified data on non-participants (*n* = 112) to establish whether there were significant demographic, diagnostic, or clinical course differences between participants and non-participants. Participants were diagnosed at hospital admission by trained raters using the Structured Clinical Interview for DSM-IV-TR (26). Diagnoses were recoded by master's level raters using DSM-5 criteria following its publication in 2013 (27). Patients in the underweight eating disorder not otherwise specified group were recoded as AN or as OSFED according to DSM-5 criteria. Non-participant diagnoses were established by chart review using DSM-5 criteria.

The participant sample was divided into short-term (illness duration <7 years; *n* = 74) and long-term ill (illness duration ≥7 years; *n* = 117) groups consistent with early definitions of SE-AN and other investigations (18, 22).

### Eating Disorder Protocol

The Johns Hopkins Eating Disorder Program includes an integrated step- down inpatient-partial hospitalization program. Treatment is delivered by a psychiatrist led, multidisciplinary team and employs a structured behavioral modification protocol described in detail elsewhere (28, 29) and in the Supplementary Material.

### Procedure

Clinical data collected for both participants and non-participants included: length of stay in the inpatient and partial hospital components of the program, admission and discharge weight, height, number of days spent on weight gain, and target weight range. Daily gowned weights were obtained by nursing staff before breakfast and after voiding.

### Clinical and Psychological Measures at Admission

Participants completed self-report questionnaires on admission, including demographic, historical, and behavioral information. Illness duration was calculated based on the question “At what age did your eating problems start to interfere with other activities?” Past intensive treatment reflective of illness severity was assessed by the number of prior eating disorder hospitalizations, general psychiatric hospitalizations, and medical hospitalizations for an eating disorder, as ascertained by the answers to the questions: “Prior to this admission, how many times have you been an inpatient on a specialized Eating Disorders Unit?”; “Prior to this admission, how many times have you been an inpatient on a general psychiatric unit (not a specialized eating disorder unit) for an eating disorder?”; “Prior to this admission, how many times have you been hospitalized on a medical unit (not a specialized eating disorder unit or general psychiatric unit) for an eating disorder?”

Three subscales of the Eating Disorders Inventory-2 were used to measure the severity of eating disorder psychopathology: drive for thinness, body dissatisfaction, and bulimia [EDI-2, (30)]. The EDI-2 is a commonly used scale that assesses eating disordered cognitions and behaviors and has good construct validity (31). Internal consistency was good to excellent (α = 0.87–0.92) in the current sample. In addition, we assessed target weight discrepancy (TWD), the difference between the patient's target weight and their desired weight. Desired weight was assessed in response to the question, “How much would you like to weigh?” Higher TWD indicates a desire to lose or maintain weight below a minimally acceptable threshold. Participants additionally completed the Beck Depression Inventory [BDI, (32)] and the NEO Five Factory Inventory [NEO-FFI, (33)]. The BDI is a widely used, 21 item self-report rating scale measuring characteristic attitudes and symptoms of depression, with good reliability and validity (34). Internal consistency for this sample was excellent (α = 0.91). The NEO-FFI is a 60-item personality inventory yielding scores in five personality domains: Neuroticism, Extraversion, Openness, Agreeableness, and Conscientiousness. The NEO-FFI has acceptable construct validity (35). Only the Neuroticism subscale was utilized in this study, as it has been positively associated with higher scores on the EDI-2, duration of illness, and length of stay (36). Internal consistency for the Neuroticism subscale in this sample was good (α = 0.87). Finally, we calculated weight suppression as the difference between the highest lifetime weight and the weight at admission (37). Weight suppression has been correlated with measures of eating disorder psychopathology (37).

### Outcomes at 6-Month Follow-Up

Participants were contacted by electronic mail 6 months after final program discharge with a link to a confidential survey asking, among other items, for their current weight. Additional assessments at follow-up included whether the patients had been rehospitalized, as well as the EDI-2 subscales and the BDI.

### Statistical Analyses

SPSS (38) and Microsoft Excel (39) software were used to perform statistical analyses. For demographic and clinical data, range, mean, standard deviation (SD), and N's are reported. Proportions are reported using raw numbers and percentages. Where short and long-term ill were compared, we employed chi-square tests for categorical variables and *t*-tests and analyses of covariance (ANCOVAs) for continuous variables, controlling for age. Repeated measures ANCOVAs were used to examine change in EDI-2 and BDI scores from admission to 6-month follow-up by illness duration group (short vs. long-term ill).

Potential predictors of BMI at 6-month follow-up were selected based on previous research indicating these variables were associated with poorer outcomes in patients with AN (9, 13, 18, 36). Bivariate correlations were used to assess whether age, clinical characteristics (admission and discharge BMI, diagnostic subtype [restricting vs. purging], length of inpatient stay, lifetime nadir BMI, total weight gained in treatment, and weight suppression), and markers of severity (illness duration, number of previous general, medical, and specialized eating disorder hospitalizations, and scores on the BDI, EDI-2, and Neuroticism subscale of the NEO-FFI) were correlated with BMI at 6-months follow-up (Supplementary Table 1). To reduce the risk of Type 1 error in this series of analyses, the threshold for statistical significance was set to *p* <.003 (.05/17 potential predictors).

The three variables that were significantly correlated with 6-month weight outcomes were entered as predictors in binary logistic regression models: admission BMI, discharge BMI, and lifetime nadir BMI. An additional variable, illness duration, was added because of strong a priori interest in this predictor, despite its lack of correlation with BMI at 6-month follow-up. The outcome variable for binary logistic regressions was coded based on BMI at 6-month follow-up, i.e., 6-month BMI of 19 kg/m^2^, entered as 0 = no, 1 = yes. Finally, to assess whether a specific BMI threshold might predict outcome, a model was built using BMI at program discharge as a predictor variable, coded based on whether or not a participant reached a discharge BMI of 19 kg/m^2^ (0 = no, 1 = yes). A binary threshold for BMI as a predictor variable was chosen because many treatment programs set a specific target weight or BMI, and based on prior literature (7, 9, 12), 19 kg/m^2^ was selected as an appropriate target BMI to examine. Alpha was set at 0.05 for the logistic regression analyses.

## Results

### Comparison of Participant and Non-participant Patients

To ascertain whether the participant sample was representative of the underweight clinical population hospitalized in the program, we compared the 191 patients who consented to participate in our full outcomes study with the 112 who declined to complete questionnaires (but from whose medical records we were permitted to abstract clinical data including demographics, diagnosis and hospital course). There were no differences in age; diagnosis; admission BMI; length of stay; total weight gained; or inpatient discharge BMI after controlling for admission BMI (all *p*'s > 0.05; Supplementary Table 2). Participants compared with non-participants gained weight more quickly [mean (SD) kg/week = 2.0 (0.87) vs. 1.8 (0.77); *p* = 0.017] and were more likely to attend the partial hospital component of treatment (74.4 vs. 51.79%; *p* < 0.001), and therefore had a slightly but significantly higher final program discharge BMI [mean (*SD*) kg/m^2^ = 20.0 (1.84) vs. 19.3 (2.26); *p* = 0.046]. These results are consistent with our previously reported findings that the effect sizes of differences between participants and non-participants in our outcomes research project are small (40).

### Baseline Characteristics

Sample characteristics are shown in Table 1. The cohort covered a wide age and BMI range, and was representative of a long-term ill sample. Mean illness duration in the long-term ill group was nearly 20 years (see Supplementary Figure 1). The mean lifetime nadir BMI for the sample as a whole was 14.4 kg/m^2^ consistent with the DSM-5 extreme range (27). More than half the sample had been previously admitted to at least one other specialty eating disorder treatment facility.

**Table 1:** Clinical and psychometric measures at admission in short-term (<7 years) and long-term (≥7 years) ill patients with anorexia nervosa.

**Clinical characteristics**	**Total sample (*****N*** **=** **191)**			**Long-term ill (*****n*** **=** **117)**		**Short-term ill (*****n*** **=** **74)**		**Sig**
	**Mean**	***SD***	**Range**	**Mean**	***SD***	**Mean**	***SD***	
Illness duration, years	13.15	11.58	(<1–53)	19.70	10.26	2.80	2.09	<0.001
Age, years	32.55	12.29	(18–73)	37.60	11.33	24.57	9.15	<0.001
Lifetime nadir BMI^a^^,^ ^b^, kg/m^2^	14.40	2.20	(8.7–20.2)	13.95	2.30	15.12	1.86	<0.001
Eating disorder hospitalizations^b^^,^ ^c^	2.35	3.64	(0–20)	3.34	4.11	0.78	1.9	<0.001
General psychiatric hospitalizations^b^	1.34	2.80	(0–20)	2.03	3.38	0.28	0.76	<0.001
Medical hospitalizations^b^	1.48	3.31	(0–20)	2.05	4.00	0.58	1.34	<0.001
Admission BMI^a^, kg/m^2^	16.21	2.10	(9.9–20.2)	16.30	1.98	16.09	2.21	0.499
**Demographics**	***N***	**%**		***N***	**%**	***N***	**%**	
Caucasian	166	88.77		106	92.98	60	82.19	0.462
**Diagnosis**^**d**^								
AN-Restricting	62	32.46		35	29.91	27	36.49	0.086
AN-Purging	104	54.45		63	53.85	41	55.41	
OSFED	25	15.06		19	17.92	6	10.00	
**Psychological measures**	**Mean**	***SD***	**Range**	**Mean**	***SD***	**Mean**	***SD***	
EDI-2 drive for thinness^e^	12.73	6.86	(0–21)	13.06	6.60	12.20	7.28	0.435
EDI-2 body dissatisfaction^e^	15.99	8.25	(0–27)	16.89	8.19	14.53	8.20	0.072
EDI-2 bulimia^e^	3.35	4.84	(0–21)	3.26	4.91	3.49	4.75	0.763
BDI depression^f^	28.97	12.94	(2–60)	31.57	12.64	25.05	12.49	0.002
NEO-neuroticism^g^	32.14	8.87	(7–48)	34.10	8.16	29.25	9.14	<0.001
Target weight discrepancy, pounds^h^	17.68	13.14	(−26–60)	19.75	12.90	14.43	12.95	0.008

a *BMI, body mass index*.

b *Analysis controlling for age*.

c *ED hospitalizations, number of prior hospitalizations on a specialty eating disorder unit*.

d *DSM-5 Diagnoses: AN, anorexia nervosa; OSFED, Other Specified Feeding and Eating Disorder*.

e *Drive for Thinness, Body Dissatisfaction, and Bulimia subscales of the Eating Disorders Inventory-2*.

f *Depression, Beck Depression Inventory, total score*.

g *Neuroticism subscale of the NEO Five Factor Inventory*.

h *Target weight discrepancy (TWD) is the difference between the participant's target weight range and her desired weight. Positive TWD expresses a desire to be thinner than is healthy*.

After controlling for age at admission, long-term ill patients reported lower lifetime nadir BMI, and higher number of specialty eating disorder, general psychiatric hospital, and medical admissions. Admission BMI did not differ between groups. For the psychological measures, EDI-2 drive for thinness, body dissatisfaction, and bulimia did not differ; however, long-term ill compared to short-term ill participants had higher neuroticism and reported greater depressive symptomatology on the BDI, with the average score of the long-term ill group falling in the “severe” range for depression, [30–63; (41)]. Long-term ill patients also had greater TWD, endorsing a desired weight farther below a medically healthy weight than did the short-term ill patients.

### Response to Hospital-Based Behavioral Weight Restoration

Patients in both groups responded well to treatment (Table 2), gaining about 2 kg per week as inpatients. Though discharge BMI, rate of weight gain, and total length of stay (inpatient plus partial hospitalization) did not differ between groups, long-term ill compared with short-term ill patients stayed nearly 8 days longer in the inpatient component of the program.

**Table 2:** Response to treatment in short-term and long-term ill patients with anorexia nervosa.

	**Long-term ill**		**Short-term ill**		**Sig**
**Treatment (*****n*** **=** **191)**					
*N*	117		74		
	**Mean**	***SD***	**Mean**	***SD***	
Inpatient length of stay, days	34.99	23.72	27.14	19.69	0.018
Total program length of stay, days	59.68	33.29	52.57	29.83	0.136
Rate of weight gain, kg/wk	2.02	1.00	1.98	0.69	0.752
Final program discharge BMI^a^	20.06	1.93	19.93	1.69	0.648
	**%**		**%**		
Discharge BMI^a^ ≥ 18	88.03		89.19		0.808
Discharge BMI^a^ ≥ 19	81.20		77.03		0.486
Discharge BMI^a^ ≥ 20	64.10		60.81		0.647
**6-month follow-up (*****n*** **=** **99)**					
*N*	63		36		
	**%**		**%**		
Response rate to follow-up questionnaire	53.85		48.65		0.484
6-month follow-up BMI^a^ ≥ 18	68.25		58.33		0.321
6-month follow-up BMI^a^ ≥ 19	58.73		52.78		0.565
6-month follow-up BMI^a^ ≥ 20	33.33		36.11		0.779
Rehospitalized within 6 months	20.63		30.56		0.268

a *BMI = body mass index, kg/m^2^*.

Despite patients' severity of illness, nearly 90% of patients attained a BMI of 18 or greater by program discharge, and four out of five patients attained a BMI of at last 19 kg/m^2^. A majority, nearly two-thirds, attained a BMI of at last 20 kg/m^2^ at program discharge.

### Outcomes at 6-Month Follow-Up

Follow-up data were available for 99 patients (52% of participants; see Table 2). There was no difference between groups (short-term vs. long-term ill) in the proportion of patients who responded to follow-up, and no difference in the proportion of patients reporting BMIs of 18, 19, or 20 kg/m^2^ at 6-month follow-up. Although most patients lost some weight after discharge, this is not unexpected and has been previously reported following intensive treatment (42, 43); however, average weight lost was <3 kg and a majority remained above a BMI of 19 kg/m^2^. Rehospitalization rate between discharge and 6-month follow-up was 24% and did not differ between groups. There was no association between attainment of a BMI ≥ 19 kg/m^2^ and rehospitalization [χ^2^_(1, N = 99)_ = 0.183, *p* = 0.669]. Follow-up BMI remained significantly higher compared to admission BMI (by at least 2.6 points; see Table 3). Measures of psychological distress including eating psychopathology and depression all decreased significantly between admission and follow-up (see Table 3).

**Table 3:** Changes in body mass index and psychological variables from admission to 6-month follow-up in short-term and long-term ill patients with anorexia nervosa.

	**Group**	**Admission**		**6-Month follow-up**		**Within-group**		**Between group**	
		**Mean**	***SD***	**Mean**	***SD***	***F***	**Sig**	***F***	**Sig**
BMI^a^	Short-term	16.30	1.98	18.92	2.24	129.842	<0.001	0.084	0.773
	Long-term	16.09	2.21	19.03	2.54				
Drive for thinness^b^	Short-term	12.20	7.28	9.92	6.66	21.441	<0.001	0.052	0.821
	Long-term	13.06	6.60	9.60	7.13				
Body dissatisfaction^b^	Short-term	14.53	8.20	14.73	8.42	4.298	0.041	0.006	0.938
	Long-term	16.89	8.19	14.57	8.09				
Bulimia^b^	Short-term	3.49	4.75	2.06	2.85	4.153	0.045	0.001	0.979
	Long-term	3.26	4.91	2.09	4.27				
Depression^c^	Short-term	25.05	12.49	20.62	12.29	12.739	0.001	0.264	0.612
	Long-term	31.57	12.64	18.86	15.62				

a *BMI = body mass index, kg/m^2^*.

b *Drive for Thinness, Body Dissatisfaction and Bulimia subscales of the Eating Disorders Inventory-2*.

c *Depression, Beck Depression Inventory, total score*.

Binary logistic regression using BMI at program discharge as a continuous predictor, along with illness duration, admission BMI, and lifetime nadir BMI, revealed that BMI at program discharge was the only significant predictor of maintaining at least a BMI of 19 kg/m^2^ at 6-month follow-up (Table 4). This was also the case when using BMI ≥ 19 kg/m^2^ as a dichotomous predictor variable. In addition, reaching a BMI of ≥ 19 kg/m^2^ at discharge was associated with 5-fold increased odds of being at a BMI of 19 kg/m^2^ at 6-month follow-up [χ^2^_(1, N = 93)_ = 5.33; *p* = 0.021, OR = 5.70].

**Table 4:** Binary logistic regression model predicting BMI ≥ 19 kg/m^2^ at 6-month follow-up using continuous discharge BMI as a predictor variable (*n* = 91).

**Predictor**	***B***	**S.E**.	**Wald**	***df***	**Sig**.	**Exp(B)**	**95% C.I. for Exp(B)**	
							**Lower**	**Upper**
Admission BMI^a^	0.070	0.170	0.173	1	0.678	1.073	0.770	1.496
Discharge BMI^a^	0.470	0.187	6.332	1	0.012	1.600	1.110	2.307
Illness duration	−0.003	0.023	0.017	1	0.897	0.997	0.954	1.042
Lifetime nadir BMI^a^	0.206	0.188	1.203	1	0.273	1.229	0.850	1.775

*The model overall is significant (chi-square = 22.30; p < 0.001)*.

a *BMI = body mass index, kg/m^2^*.

## Discussion

Experienced clinicians who treat individuals with AN inevitably encounter patients inured to treatment, who have been through multiple treatment programs, without escaping the gravitational pull of their illness. We found that, despite greater psychopathology, lower lifetime BMI, and a higher number of prior hospitalizations in the long-term ill compared to the short-term ill, the majority of long-term ill patients responded well to treatment. Short and long-term ill participants were equally likely to meet a BMI ≥ 19 kg/m^2^ by program discharge and to maintain weight at follow-up. Duration of illness was not associated with a BMI ≥ 19 kg/m^2^ at follow-up. Both groups showed sustained improvements in eating psychopathology and depressive symptomatology at 6-month follow-up, however discharge BMI was the only significant predictor of BMI at 6-month follow-up. We also found that a BMI of ≥19 kg/m^2^ at program discharge, a target met by a majority of patients in this study, was associated with a 5-fold higher likelihood of reporting a BMI ≥ 19 kg/m^2^ at 6-month follow-up.

It should be noted that follow-up data were available for 52% of participants and 32% of the entire cohort admitted during the time period examined. Outcome data on the 99 participants who responded to the outcome survey may not be representative of the full sample. While this response rate is not ideal, obtaining high response rates at follow-up from a naturalistic treatment study is challenging, especially in the U.S. where the health care system is highly fragmented. The lack of a difference in participation rate at 6-month follow-up between the short-term and long-term ill, suggests that illness chronicity did not systematically bias results for participants. Additionally, the mean discharge BMI of non-participants was also above 19 kg/m^2^; suggesting their hospital course, at least with respect to weight restoration, was similar to that of participants. We have previously shown that differences between participants and non-participants in our longitudinal treatment study are likely to exert at most small effects on outcome (40).

The integrated inpatient-partial hospitalization program described herein is designed to achieve rapid weight restoration, and we cannot exclude that some patients actively seek this aspect of treatment, however we have previously reported high levels of perceived coercion regarding hospitalization endorsed by patients at program admission with one third denying the need for hospitalization (44, 45). Despite ambivalence regarding admission, patient satisfaction with treatment at program discharge is high and may reflect therapeutic engagement and mastery over behavior change (29).

The finding that attainment of a BMI threshold of ≥19 kg/m^2^ predicts weight outcome is consistent with previous studies showing better outcomes with higher discharge weights (10, 11) and attainment of a BMI ≥19 kg/m^2^ predicting good outcome in AN (9, 12). This threshold may help explain why illness duration is commonly understood to be a poor prognostic factor. Studies frequently fail to distinguish partial from full weight restoration and vary in the definition of a good weight restoration outcome. Indeed, many studies define good outcome at 15% below ideal weight, arguably an anorectic weight (46).

In studies in which weight restoration to a BMI ≥19 kg/m^2^ is not achieved, low discharge BMI almost certainly confounds the effect of chronicity and severity. For example, in one study in which illness duration was a predictor of poor outcome, the mean discharge BMI was 15.5 kg/m^2^ (14). The same concern applies to most studies of outpatient interventions for adults with AN who often have long duration of illness and generally achieve limited weight gains (12, 18, 47).

The weight restoration rates reported here are high compared to most intensive treatment programs. For example, a recent systematic review of outcomes following residential treatment assessed nineteen open-label studies and found that only nine of these reported BMI outcomes for patients with AN (48). Of these, only one study reported mean end of treatment BMI > 18.5, corresponding with the DSM-5 diagnostic threshold for AN (49).

Time to follow-up also affects outcome. We chose to study 6-month outcome to focus on the effects of intensive treatment and avoid confounding outcome with the effects of diverse aftercare or life events that impact intermediate or longer-term risk of relapse. Six months allows for assessment of retained benefits of treatment and is consistent with data suggesting that relapse risk following inpatient weight restoration is highest in the first 3–12 months post-discharge (16). It is sobering that, even within this relatively brief follow-up period, 24% of our patients report rehospitalization, a proportion similar to the percentage who relapsed at 6-month follow-up in Carter et al.'s study (16). Discharge BMI was not however associated with likelihood of readmission in this study; reasons for readmission were not available.

The current paper joins others calling for caution in defining the construct of SE-AN. Calugi et al. (17) demonstrated that both SE-AN and non-SE-AN inpatients responded equally well to inpatient treatment, and, having attained a BMI of 19 kg/m^2^, lost a small amount of weight which was then maintained at 6- and 12-month time points. Raykos et al. (18) found that illness duration and severity of pretreatment eating psychopathology did not predict response to enhanced cognitive behavioral therapy. Wildes et al. (25) assessed the constructs underlying SE-AN in a group of patients with AN, and found that factors that most distinguished SE-AN from non-SE-AN included health-related quality of life, emotional well-being, and eating behaviors, especially binge-eating and vomiting.

The current paper extends these findings in two ways: first, by including several measures of illness severity, including state and trait psychological measures, in comparisons between short-term and long-term ill, and using measures that were correlated with outcome in a predictive model of treatment response; and second, by demonstrating that weight restoration to a BMI of at least 19 kg/m^2^ is the only significant predictor of weight outcome at 6-month follow-up. In contrast to others, purging behavior did not predict treatment outcome (16, 25).

Several study limitations in addition to the percentage of participants evaluated in follow-up require consideration.

First, weight at follow-up was self-reported. There is no evidence to suggest that illness duration would exert a bias in weight reporting, and patients with AN have been shown to provide reliable estimates of BMI, though modest (1 kg) overestimations of weight and height are frequently observed (50).

Second, the short-term 6-month follow-up interval means that participants were still potentially within the window during which risk of relapse remains relatively high (10, 16). Longer-term research assessing relapse risk is needed. Third, for reasons of statistical power, the present study was limited to adult women, and most of these were Caucasian, so it is unclear how generalizable these findings are to other socio-demographic groups.

Further research should confirm and extend understanding of the optimal threshold BMI for discharge and focus on quality of life and social and occupational functioning as indicators of illness severity, as these may have more prognostic value. That these aspects of recovery color patients' hopefulness, or lack thereof, is becoming increasingly clear. Parsing the construct of illness severity will be key to providing clinicians with the tools they need to combat the hopelessness of our most ill patients.

## Data Availability Statement

The raw data supporting the conclusions of this article will be made available by the authors, without undue reservation.

## Ethics Statement

The studies involving human participants were reviewed and approved by Johns Hopkins School of Medicine IRB. Written informed consent for participation was not required for this study in accordance with the national legislation and the institutional requirements.

## Author Contributions

GR, CS, and JC were primarily responsible for the statistical analyses. All authors contributed to the ideas in the paper, edited the drafts, and approved the submission.

## Conflict of Interest

The authors declare that the research was conducted in the absence of any commercial or financial relationships that could be construed as a potential conflict of interest.

## References

[1] Guarda AS. Treatment of anorexia nervosa: insights and obstacles. Physiol Behav. (2008) 94:113–20. 10.1016/j.physbeh.2007.11.020 18155737

[2] Agh T Kovacs G Supina D Pawaskar M Herman BK Voko Z. A systematic review of the health-related quality of life and economic burdens of anorexia nervosa, bulimia nervosa, and binge eating disorder. Eat Weight Disord. (2016) 21:353–64. 10.1007/s40519-016-0264-x 26942768 PMC5010619

[3] Arkell J Robinson P. A pilot case series using qualitative and quantitative methods: biological, psychological and social outcome in severe and enduring eating disorder (anorexia nervosa). Int J Eat Disord. (2008) 41:650–6. 10.1002/eat.20546 18446832

[4] Yager J. Managing patients with severe and enduring anorexia nervosa: when is enough, enough? J Nerv Ment Dis. (2020) 208:277–82. 10.1097/NMD.0000000000001124 32221180

[5] Eddy KT Tabri N Thomas JJ Murray HB Keshaviah A Hastings E. Recovery from anorexia nervosa and bulimia nervosa at 22-year follow-up. J Clin Psychiatry. (2017) 78:184–9. 10.4088/JCP.15m10393 28002660 PMC7883487

[6] Fichter MM Quadflieg N Crosby RD Koch S. Long-term outcome of anorexia nervosa: results from a large clinical longitudinal study. Int J Eat Disord. (2017) 50:1018–30. 10.1002/eat.22736 28644530

[7] Khalsa SS Portnoff LC McCurdy-McKinnon D Feusner JD. What happens after treatment? A systematic review of relapse, remission, and recovery in anorexia nervosa. J Eat Disord. (2017) 5:20. 10.1186/s40337-017-0145-3 28630708 PMC5470198

[8] Bardone-Cone AM Hunt RA Watson HJ. An overview of conceptualizations of eating disorder recovery, recent findings, and future directions. Curr Psychiatry Rep. (2018) 20:79. 10.1007/s11920-018-0932-9 30094740

[9] Kaplan AS Walsh BT Olmsted M Attia E Carter JC Devlin MJ. The slippery slope: prediction of successful weight maintenance in anorexia nervosa. Psychol Med. (2009) 39:1037–45. 10.1017/S003329170800442X 18845008 PMC4449142

[10] Rigaud D Pennacchio H Bizeul C Reveillard V Verges B. Outcome in AN adult patients: a 13-year follow-up in 484 patients. Diabetes Metab. (2011) 37:305–11. 10.1016/j.diabet.2010.11.020 21317006

[11] El Ghoch M Calugi S Chignola E Bazzani PV Dalle Grave R. Body mass index, body fat and risk factor of relapse in anorexia nervosa. Eur J Clin Nutr. (2016) 70:194–8. 10.1038/ejcn.2015.164 26419195

[12] Lock J Agras WS Le Grange D Couturier J Safer D Bryson SW. Do end of treatment assessments predict outcome at follow-up in eating disorders? Int J Eat Disord. (2013) 46:771–8. 10.1002/eat.22175 23946139

[13] Vall E Wade TD. Predictors of treatment outcome in individuals with eating disorders: a systematic review and meta-analysis. Int J Eat Disord. (2015) 48:946–71. 10.1002/eat.22411 26171853

[14] Fichter MM Quadflieg N Hedlund S. Twelve-year course and outcome predictors of anorexia nervosa. Int J Eat Disord. (2006) 39:87–100. 10.1002/eat.20215 16231345

[15] Clausen L. Time to remission for eating disorder patients: a 2(1/2)-year follow-up study of outcome and predictors. Nord J Psychiatry. (2008) 62:151–9. 10.1080/08039480801984875 18569780

[16] Carter JC Mercer-Lynn KB Norwood SJ Bewell-Weiss CV Crosby RD Woodside DB. A prospective study of predictors of relapse in anorexia nervosa: implications for relapse prevention. Psychiatry Res. (2012) 200:518–23. 10.1016/j.psychres.2012.04.037 22657951

[17] Calugi S El Ghoch M Dalle Grave R. Intensive enhanced cognitive behavioural therapy for severe and enduring anorexia nervosa: a longitudinal outcome study. Behav Res Ther. (2017) 89:41–8. 10.1016/j.brat.2016.11.006 27863331

[18] Raykos BC Erceg-Hurn DM McEvoy PM Fursland A Waller G. Severe and enduring anorexia nervosa? Illness severity and duration are unrelated to outcomes from cognitive behaviour therapy. J Consult Clin Psychol. (2018) 86:702–9. 10.1037/ccp0000319 30035586

[19] Maguire S Touyz S Surgenor L Crosby RD Engel SG Lacey H. The clinician administered staging instrument for anorexia nervosa: development and psychometric properties. Int J Eat Disord. (2012) 45:390–9. 10.1002/eat.20951 22407867 PMC8674751

[20] Hay P Touyz S. Classification challenges in the field of eating disorders: can severe and enduring anorexia nervosa be better defined? J Eat Disord. (2018) 6:41. 10.1186/s40337-018-0229-8 30555695 PMC6287358

[21] Wonderlich SA Bulik CM Schmidt U Steiger H Hoek HW. Severe and enduring anorexia nervosa: update and observations about the current clinical reality. Int J Eat Disord. (2020) 53:1303–12. 10.1002/eat.23283 32359125

[22] Touyz S Le Grange D Lacey H Hay P Smith R Maguire S. Treating severe and enduring anorexia nervosa: a randomized controlled trial. Psychol Med. (2013) 43:2501–11. 10.1017/S0033291713000949 23642330

[23] Broomfield C Stedal K Touyz S Rhodes P. Labeling and defining severe and enduring anorexia nervosa: a systematic review and critical analysis. Int J Eat Disord. (2017) 50:611–23. 10.1002/eat.22715 28444828

[24] Williams KD Dobney T Geller J. Setting the eating disorder aside: an alternative model of care. Eur Eat Disord Rev. (2010) 18:90–6. 10.1002/erv.989 20099264

[25] Wildes JE Forbush KT Hagan KE Marcus MD Attia E Gianini LM. Characterizing severe and enduring anorexia nervosa: an empirical approach. Int J Eat Disord. (2017) 50:389–97. 10.1002/eat.22651 27991694 PMC5386793

[26] American Psychiatric Association. Diagnostic and Statistical Manual of Mental Disorders. Washington, DC: American Psychiatric Press (1994).

[27] American Psychiatric Association DSM-5 Task Force. Diagnostic and Statistical Manual of Mental Disorders: DSM-5. Washington, DC: American Psychiatric Association (2013). 10.1176/appi.books.9780890425596

[28] Redgrave GW Coughlin JW Schreyer CC Martin LM Leonpacher AK Seide M. Refeeding and weight restoration outcomes in anorexia nervosa: challenging current guidelines. Int J Eat Disord. (2015) 48:866–73. 10.1002/eat.22390 25625572

[29] Guarda AS Cooper M Pletch A Laddaran L Redgrave GW Schreyer CC. Acceptability and tolerability of a meal-based, rapid refeeding, behavioral weight restoration protocol for anorexia nervosa. Int J Eat Disord. (2020) 53:2032–7. 10.1002/eat.23386 33026118

[30] Garner DM. Eating Disorders Inventory-2 Professional Manual. Odessa, FL: Psychological Assessment Resources, Inc. (1991).

[31] Espelage DL Mazzeo SE Aggen SH Quittner AL Sherman R Thompson R. Examining the construct validity of the Eating Disorder Inventory. Psychol Assess. (2003) 15:71–80. 10.1037/1040-3590.15.1.71 12674726

[32] Beck AT Ward CH Mendelson M Mock J Erbaugh J. An inventory for measuring depression. Arch Gen Psychiatry. (1961) 4:561–71. 10.1001/archpsyc.1961.01710120031004 13688369

[33] Costa PT McCrae RR. The Manual for the NEO Five Factor Inventory. Odessa, FL: Psychological Assessment Resources (1992).

[34] Schotte CK Maes M Cluydts R De Doncker D Cosyns P. Construct validity of the Beck Depression Inventory in a depressive population. J Affect Disord. (1997) 46:115–25. 10.1016/S0165-0327(97)00094-3 9479615

[35] Holden RR Fekken GC. The NEO Five-Factor Inventory in a Canadian context: psychometric properties for a sample of university women. Pers. Individ. Differ. (1994) 17:441. 10.1016/0191-8869(94)90291-7

[36] Fischer LK Schreyer CC Coughlin JW Redgrave GW Guarda AS. Neuroticism and clinical course of weight restoration in a meal-based, rapid-weight gain, inpatient-partial hospitalization program for eating disorders. Eat Disord. (2017) 25:52–64. 10.1080/10640266.2016.1241056 27775490

[37] Lowe MR Piers AD Benson L. Weight suppression in eating disorders: a research and conceptual update. Curr Psychiatry Rep. (2018) 20:80. 10.1007/s11920-018-0955-2 30155651

[38] IBM Corp. IBM SPSS Statistic for Windows. Amonk, NY: IBM Corp. (2011).

[39] Microsoft Corporation. Microsoft Excel for Mac 2008. Redmond, WA: Microsoft Corporation (2007).

[40] Schreyer CC Redgrave GW Hansen JL Guarda AS. Self-selection bias in eating disorders outcomes research: Does treatment response of underweight research participants and non-participants differ? Int J Eat Disord. (2017) 50:602–5. 10.1002/eat.22650 28225563

[41] Beck AT Steer RA Garbin MG. Psychometric properties of the Beck Depression Inventory: twenty-five years of evaluation. Clin Psychol Rev. (1988) 8:77–100. 10.1016/0272-7358(88)90050-5

[42] Russell GF Szmukler GI Dare C Eisler I. An evaluation of family therapy in anorexia nervosa and bulimia nervosa. Arch Gen Psychiatry. (1987) 44:1047–56. 10.1001/archpsyc.1987.01800240021004 3318754

[43] Dalle Grave R Calugi S Conti M Doll H Fairburn CG. Inpatient cognitive behaviour therapy for anorexia nervosa: a randomized controlled trial. Psychother Psychosom. (2013) 82:390–8. 10.1159/000350058 24060628 PMC3884188

[44] Guarda AS Pinto AM Coughlin JW Hussain S Haug NA Heinberg LJ. Perceived coercion and change in perceived need for admission in patients hospitalized for eating disorders. Am J Psychiatry. (2007) 164:108–14. 10.1176/ajp.2007.164.1.108 17202551

[45] Schreyer CC Coughlin JW Makhzoumi SH Redgrave GW Hansen JL Guarda AS. Perceived coercion in inpatients with anorexia nervosa: Associations with illness severity and hospital course. Int J Eat Disord. (2016) 49:407–12. 10.1002/eat.22476 26578421

[46] Strober M Freeman R Morrell W. The long-term course of severe anorexia nervosa in adolescents: survival analysis of recovery, relapse, and outcome predictors over 10–15 years in a prospective study. Int J Eat Disord. (1997) 22:339–60. 10.1002/(SICI)1098-108X(199712)22:4<339::AID-EAT1>3.0.CO;2-N 9356884

[47] Hay PJ Touyz S Sud R. Treatment for severe and enduring anorexia nervosa: a review. Aust N Z J Psychiatry. (2012) 46:1136–44. 10.1177/0004867412450469 22696548

[48] Peckmezian T Paxton SJ. A systematic review of outcomes following residential treatment for eating disorders. Eur Eat Disord Rev. (2020) 28:246–59. 10.1002/erv.2733 32196843 PMC7216912

[49] Twohig MP Bluett EJ Cullum JL Mitchell PR Powers PS Lensegrav-Benson T. Effectiveness and clinical response rates of a residential eating disorders facility. Eat Disord. (2016) 24:224–39. 10.1080/10640266.2015.1064279 26214231

[50] Ciarapica D Mauro B Zaccaria M Cannella C Polito A. Validity of self-reported body weight and height among women including patients with eating disorders. Eat Weight Disord. (2010) 15:e74–80. 10.1007/BF03325282 20571324

