# Supplementary material for: Discharge Body Mass Index, Not Illness Chronicity, Predicts 6-Month Weight Outcome in Patients Hospitalized With Anorexia Nervosa
Source: Front Psychiatry. 2021 Feb 25;12:641861. doi: 10.3389/fpsyt.2021.641861 (PMC7946839; doi:10.3389/fpsyt.2021.641861)
Supplement: Supplementary file 1 [file Table_1.docx]

Supplemental Table 1. Bivariate correlations of possible predictor variables and BMI at 6-month follow-up

| Possible Predictor Variable | BMI at 6 month follow-up (Continuous^c^) | BMI at 6 month follow-up > 19.0 kg/m^2^ (yes/no) |
| --- | --- | --- |
| Age (years) | 0.05 | 0.04 |
| Clinical Characteristics |  |  |
| Admission BMI, kg/m^2^ | 0.43* | 0.34* |
| Discharge BMI, kg/m^2^ | 0.57* | 0.45* |
| Total weight gained, kg | 0.10 | 0.08 |
| Weight suppression, kg | 0.18 | 0.10 |
| Diagnostic subtype^a^ | 0.13 | 0.06 |
| Length of inpatient stay, days | -0.09 | -0.06 |
| Markers of Severity |  |  |
| Lifetime nadir BMI, kg/m^2^ | 0.51* | 0.37* |
| Illness duration, years | -0.10 | -0.05 |
| General psychiatric admissions^b^ | 0.04 | -0.04 |
| Medical admissions^b^ | -0.06 | -0.12 |
| Specialized eating disorder admissions^b^ | -0.15 | -0.22 |
| BDI score | -0.04 | 0.001 |
| EDI-2 Bulimia score | -0.06 | -0.15 |
| EDI-2 Body Dissatisfaction score | 0.05 | 0.10 |
| EDI-2 Drive for Thinness score | -0.12 | -0.002 |
| NEO-FFI Neuroticism score | -0.11 | -0.06 |
| Abbreviations: BMI = Body Mass Index, BDI = Beck Depression Inventory, EDI-2 = Eating Disorder Inventory, 2^nd^ Edition.  **p* < .003 (adjusted alpha level for multiple comparisons).  ^a^Diagnostic subtype refers to the restricting and binge-purge subtypes of anorexia nervosa.  ^b^Measured by number of admissions.  ^c^Variable was continuous, rather than dichotomous. | | |
