# Supplementary material for: Discharge Body Mass Index, Not Illness Chronicity, Predicts 6-Month Weight Outcome in Patients Hospitalized With Anorexia Nervosa
Source: Front Psychiatry. 2021 Feb 25;12:641861. doi: 10.3389/fpsyt.2021.641861 (PMC7946839; doi:10.3389/fpsyt.2021.641861)
Supplement: Supplementary file 2 [file Table_2.docx]

| Supplemental Table 2. Comparison of Participants and Non-participants on Age and Clinical Characteristics   \|  \| Participants  *M* (*SD*) \| Non-Participants  *M* (*SD*) \| *t/F* (Effect size^b^) \| \| --- \| --- \| --- \| --- \| \| Age, years \| 32.55 (12.29) \| 34.64 (14.34) \| 1.34 (.16) \| \| Admission BMI, kg/m^2^ \| 16.17 (2.12) \| 15.71 (2.34) \| -1.75 (.20) \| \| Inpatient rate of weight gain, kg/week \| 1.99 (0.87) \| 1.75 (0.78) \| -2.41* (.28) \| \| Inpatient discharge BMI^a^, kg/m^2^ \| 19.02 (1.82) \| 18.56 (1.94) \| 1.60 (.005) \| \| Length of stay, days \| 56.92 (32.10) \| 49.78 (34.92) \| -1.80 (.21) \| \| Total weight gained, kg \| 22.51 (11.42) \| 20.34 (14.44) \| -1.35 (.17) \| \| Final discharge BMI^a^, kg/m^2^ \| 20.01 (1.84) \| 19.25 (2.26) \| 6.98* (.02) \| \|  \| % \| % \| *χ^2^* (Phi) \| \| Diagnosis \|  \|  \| 3.01 (.10) \| \| AN-R \| 32.46 \| 41.07 \|  \| \| AN-BP \| 54.45 \| 47.32 \|  \| \| OSFED \| 13.09 \| 11.61 \|  \| \| Partial Hospital Attendance \| 74.35 \| 51.79 \| 16.01* (.23) \| \| Abbreviations: BMI = Body Mass Index, AN-R = Anorexia nervosa, restricting subtype, AN-BP = Anorexia Nervosa, Binge-Purge subtype  **p* < .05.  ^a^Controlling for admission BMI.  ^b^Effect sizes were Cohen’s D for independent t-test analyses, ƞ_p_^2^ (partial eta squared) for analyses of covariance, and phi for chi-square analyses. \| \| \| \| |
| --- | --- | --- | --- | --- | --- | --- | --- | --- | --- | --- | --- | --- | --- | --- | --- | --- | --- | --- | --- | --- | --- | --- | --- | --- | --- | --- | --- | --- | --- | --- | --- | --- | --- | --- | --- | --- | --- | --- | --- | --- | --- | --- | --- | --- | --- | --- | --- | --- | --- | --- | --- | --- | --- | --- | --- | --- | --- | --- | --- | --- |
