# Supplementary figures and images for: Discharge Body Mass Index, Not Illness Chronicity, Predicts 6-Month Weight Outcome in Patients Hospitalized With Anorexia Nervosa
Source: Front Psychiatry. 2021 Feb 25;12:641861. doi: 10.3389/fpsyt.2021.641861 (PMC7946839; doi:10.3389/fpsyt.2021.641861)

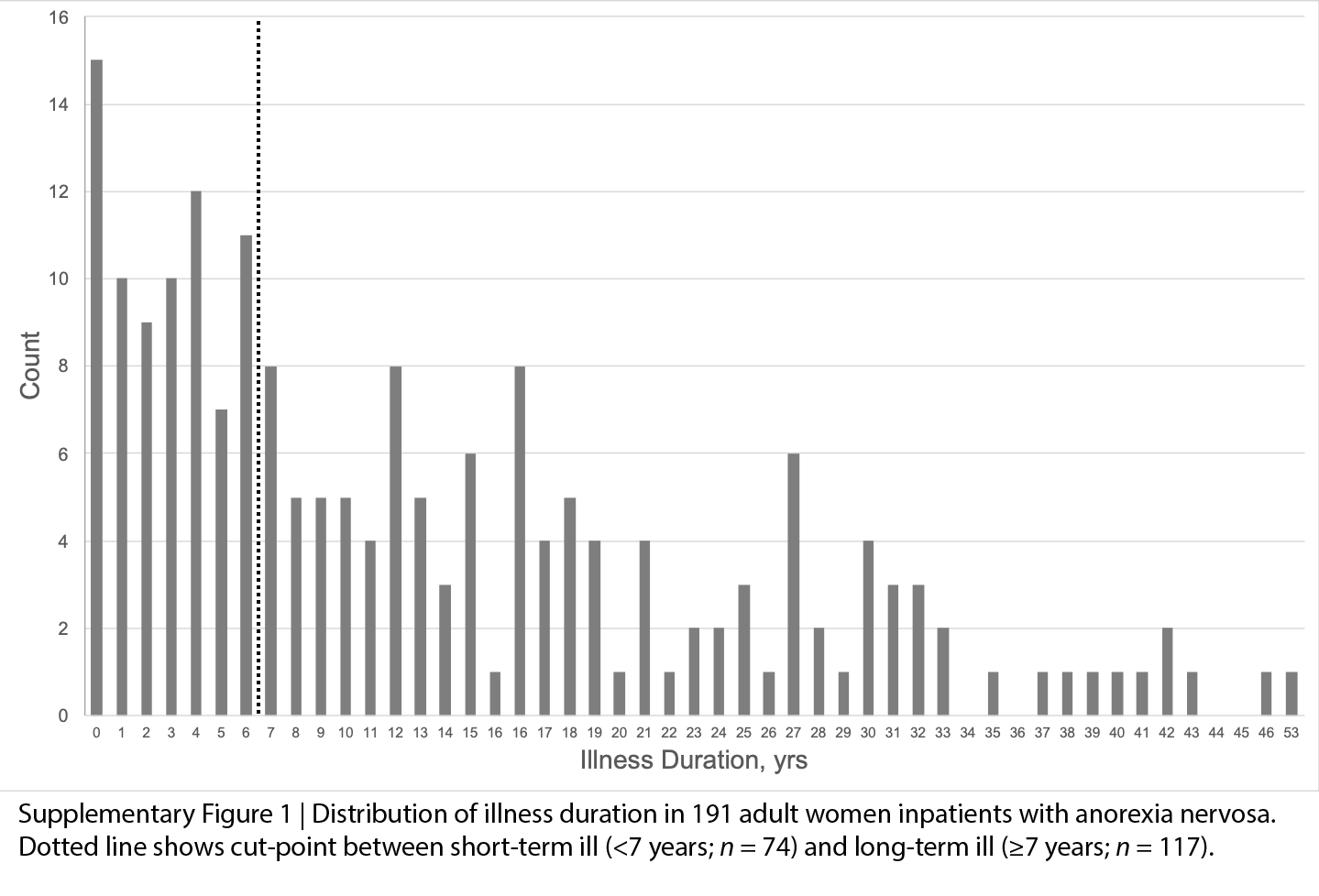

Supplement: Supplementary file 3 [file Image_1.tif]
